# Post-traumatic growth in polytraumatized patients after 20+ years: a long-term follow-up study of 337 patients treated at a level 1 trauma center

**DOI:** 10.1007/s00068-022-02022-w

**Published:** 2022-06-28

**Authors:** Yannik Kalbas, Sascha Halvachizadeh, Yohei Kumabe, Anna Theresa Luidl, Jennifer Lynne Steel, Boris A. Zelle, Paolo Cinelli, Hans-Christoph Pape, Roman Pfeifer

**Affiliations:** 1 Department of Trauma Surgery and Harald-Tscherne Laboratory, University Hospital Zurich, University of Zurich, Ramistr. 100, 8091 Zurich, Switzerland; 2 Faculty of Medicine, RWTH Aachen University, Aachen, Germany; 3 Department of Surgery, University of Pittsburgh School of Medicine, Pittsburgh, PA USA; 4 Department of Orthopaedics, UT Health San Antonio, Joe R. & Teresa Lozano Long School of Medicine, 7703 Floyd Curl Drive, San Antonio, TX 78229 USA

**Keywords:** Post-traumatic growth, Polytrauma, Long-term follow-up, Outcome, Coping

## Abstract

**Purpose:**

There is limited research on the long-term psychiatric outcomes of polytraumatized patients. Existing studies focus mainly on the negative sequelae. Post-traumatic growth (PTG) describes positive personal development after severe physical or mental distress. In this study, we investigated post-traumatic growth in polytraumatized patients at least 20 years after trauma.

**Methods:**

Patients treated for polytrauma at a German level 1 trauma center between 1971 and 1990, were contacted 20+ years later. A questionnaire with 37 questions from the stress-related growth scale (SRGS) and the post-traumatic growth inventory (PGI) was administered. PTG was quantified in five specific areas. PTG and patient demographics were then analyzed using logistic regression.

**Results:**

Eligible questionnaires were returned by 337 patients. 96.5% of patients reported improvements regarding at least one of the 37 questions. Approximately, a third of patients noticed distinct improvements regarding their relationship to others (29.2%), appreciation of life (36.2%) and attitudes towards new possibilities (32.5%). Patient demographics were significant predictors for the development of PTG: Older (*p* < 0.001), female (*p* = 0.042) and married patients (*p* = 0.047) showed a greater expression of PTG. We also saw significantly more PTG in patients with higher injury severity (*p* = 0.033).

**Conclusion:**

20 years after polytrauma, patients report improvements in their relationship with others, appreciation of life and attitude towards new possibilities. Women and married patients show higher expression of PTG. Furthermore, there is higher expression of PTG with higher age and injury severity. Post-traumatic growth should be identified and fostered in clinical practice.

**Level of evidence:**

III—prospective long-term follow-up study.

## Purpose

Injury remains amongst the leading causes of death, especially in young adults [1, 2]. High impact traumas such as vehicular traffic accidents, falls from height and industrial accidents are associated with multiple injuries and negative outcomes [3]. Through the adaptation and standardization of treatment algorithms, increased use of diagnostic tools and the improvement of (road) safety precautions, complications and mortality of polytraumatized patients have decreased significantly [4, 5]. This, in turn, led to an increase in long-term consequences in survivors, such as reductions in quality of life [6, 7].

Previous studies from our group have investigated the long-term socio-economic outcomes and psychosocial sequelae of polytrauma in a cohort from a German level one trauma center [6–12]. Functional impairment, disability, chronic pain, unemployment, financial deficits and psychological sequelae were common among these survivors of polytrauma [11]. Long-term psychiatric outcomes after polytrauma include post-traumatic stress disorder, depression and anxiety, which interfere with rehabilitation and return to self-sufficiency [9, 10].

Individuals, who experience severe stress, however, can also show signs of positive personal development, which is referred to as post-traumatic growth [13]. It was first described in 1964 and has been studied in survivors of natural disasters, interpersonal violence, cancer, and veterans [13–16]. Research on post-traumatic growth in the field of physical injury, however, is limited and often focuses on patients with physical disability or investigates small patient cohorts over shorter periods of time [17, 18]. Understanding the manifestation of long-term post-traumatic growth in survivors of polytrauma might show considerable potential for rehabilitation.

In this long-term cohort study, we performed a survey of polytraumatized patients 20 or more years after treatment at the same level one trauma center. The aims of this study were to determine the following:

- At what rate does post-traumatic growth manifest in patients after polytrauma?
- What are the predictors of post-traumatic growth in survivors of polytrauma?

## Methods

The reporting of this study is adhering to the STROBE statement [19].

### Ethical considerations

This study was approved by the local ethics committee of the Hannover Medical School and conducted according to the Declaration of Helsinki [20]. Only patients who consented to participate in this study were included. (Ethical Committee Trial ID Number 2325-200/03/22).

### Study population

We contacted 631 polytraumatized patients, who were injured between January 1, 1973 and December 31, 1990 and received treatment at the Hannover University Hospital, a German level 1 trauma center. Patients belong to a previously established database for a follow-up study aiming at psychosocial long-term effects [8, 11, 12]. Inclusion criteria were an age between 3 and 60 years at the time of injury and written consent. Exclusion criteria were amputations of the upper and lower extremity and paraplegia.

### Recruitment

Patients were contacted by phone to verify current addresses and consent to participate. If patients could not be reached through the information on record, local registration- or post offices were contacted. Self-administered questionnaires were sent out, if patients agreed to participate.

### Definitions

Polytrauma was defined as at least two long bone fractures, or one life-threatening injury and at least one additional injury, or severe head trauma and at least on additional injury. This definition by Tscherne in 1966 was the current standard at the time the database was established [21]. The degree of injury was categorized by the Injury Severity Score (ISS) [22].

Post-traumatic growth describes perceived changes in an individual’s attitude or worldview as response to an intense stressor such as pain, physical restrictions or psychological trauma [23, 24]. Through understanding, handling, and accepting the situation, it is possible to reintegrating a traumatic event into one’s personal experience and achieve personal growth in areas such as appreciation of life, relationship with others or realization of one’s potential [23, 24].

### Questionnaire

Patients received a self-administered questionnaire, with German translations of the “post-traumatic growth inventory” (PTGI) and the a shortened version of the “stress-related growth scale” (SRGS) [25, 26]. Both translations were validated with strong internal consistency and a coefficient alpha of *α* = 0.91 and *α* = 0.92 respectively [27]. A total of 37 questions were asked which inquired about five categories, as proposed by the PTGI: Relationships to others (11 of 37), personal strengths (12 of 37), appreciation of life (9 of 37), new possibilities (3 of 37) and spiritual change (2 of 37). Each question aimed at quantifying subjective improvements regarding specific aspects. Answers were scored on a Likert scale from 1 to 3: “No improvements” (1). “Some improvements” (2). A lot of improvements” (3) and scores were averaged amongst categories and among patients. Patients who completed all 37 questions were then stratified into three groups according to their overall expression of post-traumatic growth (111–87: high; 86–62: some; 61–37: very little to none).

### Statistics

Continuous data are presented with mean (± Standard Deviation. SD), categorical variables with numbers and percentages. Odds ratios were calculated based on multivariable logistic regression model. Outcome variable was the expression of post-traumatic growth as measured by the score obtained from the questionnaire. Dependent variables included injury severity (ISS), length of stay (LOS) and length of ICU stay (ICU_LOS), and non-injury-related factors (gender, marital status and age at injury), as these variables have known clinical influence. The variables in the model were assumed to be independent. The statistical significance level was set at *p* < 0.05. Statistical analysis was performed using R (R Core Team (2018). R: A language and environment for statistical computing. R Foundation for Statistical Computing, Vienna, Austria. URL: https://www.R-project.org/).

## Results

### Demographics

**Fig. 1:**
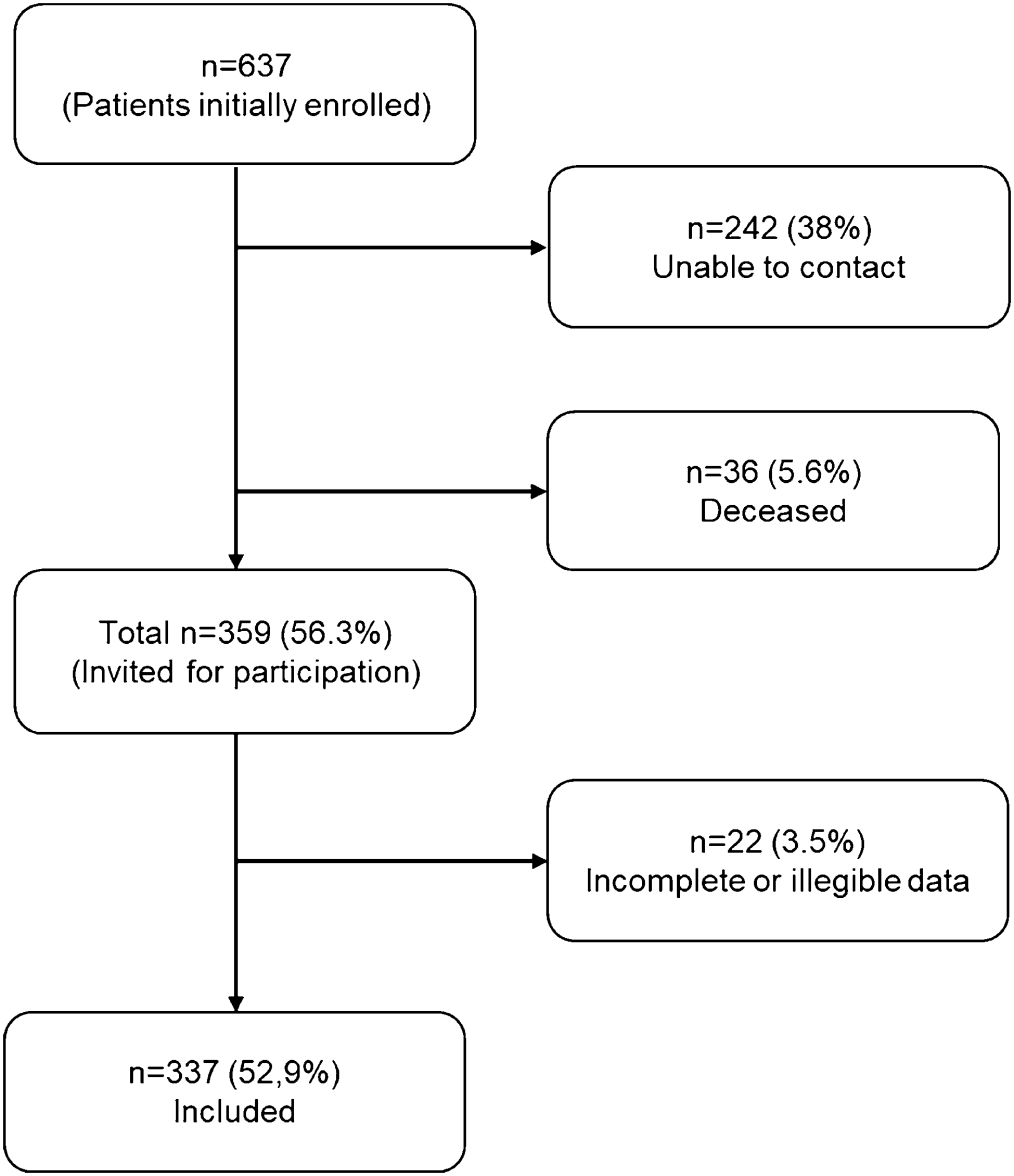
Patient inclusion flowchart: initially, 637 patients were included in the primary study population. Of these, 242 (38%) patients could not be contacted and 36 (5.6%) patients died. Of 359 patients invited to participate, 22 (3.5%) patients returned an incomplete or illegible questionnaire, leaving 337 (52.9%) patients who were enrolled in this study. *n* number

Of 637 patients initially enrolled, 337 (52.9%) returned an eligible questionnaire and were included. A total of 36 (5.6%) patients had deceased and 242 (38%) were unable to contact. Further 21 questionnaires (3.3%) were incomplete and one (0.2%) had illegible information and were not included in the analyses. Recruitment process is visualized in Fig. 1. Demographics are summarized in Table 1. Of the 337 patients included, gender distribution was 114 (33.8%) female to 223 (66.2%) male. Mean age at time of the accident was 25.4 years (SD = 11.7) and median ISS was 19 (mean = 20.3, SD = 9.3). Average age at follow-up was 52 years (SD = 11.76) and median time since injury was 28 years (SD = 4.92). Patients who were not included (*n* = 300) showed no significant differences concerning demographics.

**Table 1:** Patient demographics

*n*	337
Age at injury in years [mean (SD)]	25.4 (11.7)
Age at follow-up in years [mean (SD)]	52 (11.76)
Time since injury in years [median (SD)]	28 (4.92)
Gender, female (%)	114 (33.8)
Married *n* (%)	107 (35.8)
ISS points [median/mean (SD)]	19 / 20.3 (9.3)
Length of stay ICU in days [mean (SD)]	12.3 (11.1)
Length of stay in days [mean (SD)]	29.8 (23.4)

Values in mean (SD) and percentage

### Rate of post-traumatic growth in polytrauma survivors

Overall, only 15% of patients show very little to no expression of post-traumatic growth while 20% of patients rated post-traumatic growth as high. Tables 2, 3 show the distribution of answers by categories and the five aspects most frequently rated positively: Overall, 96.5% of patients described subjective improvements in at least one of the 37 aspects. Most positive changes were reported regarding appreciation of life (36.2%) with “not taking health for granted” (54.8%) and “realizing the worth of health” (43.7%) as the questions with the most positive answers. A third of patients registered a more positive attitude towards new possibilities (32.5%) and 29.2% of patients described improvements in their perceived relationships to others, especially regarding “confidence in others in time of need” (43.3%). Patients saw fewer improvements regarding their perceived personal strengths (23.4%) and their attitude towards religion and faith (20.5%).

**Table 2:** Distribution of PTG domains by number of positive answers in percent

Category	A lot (%)	Some (%)	None (%)
Appreciation of life	36.2	41.1	22.7
New possibilities	32.4	41.6	25.9
Relationships to others	29.2	40.6	30.2
Personal strengths	23.4	41.6	35.0
Spiritual change	20.5	23.8	55.7

**Table 3:** Five questions most frequently answered positively in percent

Aspect	Domain	A lot (%)	Some (%)	None (%)
Not taking health for granted	Appreciation of life	54.8	35.4	9.8
Realizing the worth of life	Appreciation of life	43.7	33.2	23.1
Confidence in others in times of need	Relationships to others	43.3	39.9	16.9
Gratitude	Appreciation of life	40.7	42.5	16.8
Seeing the positive	Appreciation of life	38.4	40.5	21.0

### Predictors of PTG in polytrauma survivors

Results from regression analysis are summarized in Table 4. Age at injury was significantly (*p* < 0.001) lower in patients with no expression of post-traumatic growth compared to the other two groups. Patients reporting high expression had a higher age at injury [27.9 years vs. 26.8 years (some) and 19.8 years (none)]. We also see significant differences between groups when comparing gender (*p* = 0.042) and marital status (*p* = 0.047): The percentage of females is highest (36.8%) in patients with high expression and lowest (14.3%) in patients with no expression of post-traumatic growth. Nearly, half (47.4%) of patients with high expression are married, while only a quarter (23.2%) of those with no expression are. Furthermore, we noticed that patients who describe higher expression had on average a higher ISS (23.5) than those with no expression (18.3). These findings were also significant (*p* = 0.033). We found no connection between expressions of post-traumatic growth and the overall length of stay (LOS) or the LOS in the Intensive Care Unit (ICU_LOS).

**Table 4:** Results from regression analysis: patients are stratified by expression of post-traumatic growth

	High	Some	None	*P* value
*n*	44 (20.8%)	188 (64.2%)	61 (15.0%)	
Age at injury [mean (SD)]	27.9 (11.3)	26.8 (11.6)	19.8 (10.4)	< 0.001
Gender, female (%)	36.8	24.7	14.3	0.042
Marital status, married (%)	18 (47.4)	62 (36.5)	13 (23.2)	0.047
ISS [mean (SD)]	23.5 (8.9)	20.5 (9.5)	18.3 (9.6)	0.033
ICU_LOS [mean (SD)]	12.16 (9.1)	13.4 (12.5)	12.2 (8.1)	0.803
LOS [mean (SD)]	30.0 (18.7)	29.63 (21.4)	26.1 (24.9)	0.649

Values in mean (SD) and percentage

A *p* value of < 0.05 is deemed significant

## Discussion

Surviving a polytrauma is a devastating experience that leaves people restricted in multiple areas of their daily lives [6, 7, 11, 28]. The long-term psychological response to such severe physical injury, however, is not exclusively negative. Through our study, we were able to gain insight into long-term post-traumatic growth after polytrauma and made the following discoveries:

- Many severely injured patients express post-traumatic growth and show subjective improvements in at least one domain of post-traumatic growth.
- Positive predictors of post-traumatic growth included being female, married, older, and having greater injury severity.

## Expression of post-traumatic growth

The expression of post-traumatic growth is positively influenced by optimism and social support as well as resilience, social status, psychological health, education and self-efficacy [23, 24, 29]. These factors also serve a predictors for outcome, rehabilitation potential, and return to work after severe physical injury [30, 31].

Our findings suggest potential for post-traumatic growth in many polytraumatized patients; therefore, identifying and fostering it might prove beneficial for outcome and emotional well-being.

While most previously conducted studies on post-traumatic growth were performed in the fields of cancer, interpersonal violence, and natural disasters, the literature on post-traumatic growth after physical injury is very limited [14–16, 24, 29, 32–34]. Comparing physical injury to those fields, there are notable overlaps considering physiological stress, yet there are differences in disability, employment, and chronic pain. As this plays an important role in integrating our findings in the scientific context, each finding will be discussed independently in regards to post-traumatic growth and the outcome after polytrauma.

## Influence of gender, marital status, injury severity and age

Our findings on the role of gender concur with the previously published literature [24]. A meta-analysis of 70 studies and 16,076 patients showed a moderate effect of female gender on the expression of post-traumatic growth [32]. One possible reason for this finding is the fact that women engage in more deliberate ruminating thoughts, which can encourage reflection, increases awareness and has been shown to positively influence post-traumatic growth [24] [35]. Women also tend to utilize a more emotion-focused coping style, which is linked to greater expression of post-traumatic growth [24, 36]. In the field of physical injuries, our results are unexpectedly positive, as previous studies have shown that women tend to have worse functional outcome after major trauma, as well as a reduced quality of life, and higher incidence of depression [37, 38]. Such findings, however, are often limited by short observation periods [37]. One previous study from our group showed no significant gender-specific difference in the expression of negative psychological sequelae after 20 or more years [10]. Furthermore, another study of our cohort showed no gender-specific differences in financial, social and medical impairments between 10 and 20 years post-injury [12]. Combined, these findings suggest that the female’s approach in dealing with trauma results in the report of post-traumatic growth even years after the injury.

While there are some conflicting views on the role of marital status on post-traumatic growth, there is a clear concurrence on the beneficial role of social support [29]. In the field of accidental injuries, one study shows an association of between marital status and post-traumatic growth 18 months post-injury, matching with our results [39]. One of the aspects of our questionnaire with the most positive answers concerned “confidence in others in times of need” and about a third of patients described positive changes regarding their “relationship to others”. This concurs with other studies, which reported long lasting improvements in close relationships through post-traumatic growth [40]. As an instantaneous loss of independence, which often comes with severe injury, requires physical and emotional support, one might be more likely to develop trust in others, resulting in such lasting improvements.

Being older at the time of injury also resulted in greater levels of post-traumatic growth. Regarding acquired physical disability, a study from 2019 reported older age as a positive influence within the first year after rehabilitation [41]. This corresponds with our results regarding post-traumatic growth. A previous study from our group found younger patients suffered more severe social restrictions (reduction in number of friends) than older patients [6]. We ascribe this to long hospital stays and a long rehabilitation process limiting the contact with peers. Additionally, functional deficits or cognitive impairment might lead to exclusion from leisure activities, which is further aggravated by failing classes or having to change schools [42]. Our previous studies also showed that younger patients have more frequent financial net income losses and are more often in debt [6]. This is likely due to disability and chronic pain, which have been shown to negatively influence on socio-economic and employment status [30, 31]. Overall, younger patients are more likely to suffer financial and social consequences after a severe injury, which decrease overall contentment.

We observed higher rates of post-traumatic growth in patients that were more severely injured. Considering the socio-economic deficits and reduced quality of life associated with severe injury, these results seem counterintuitive [6, 7, 11, 28]. However, our findings concur with results from literature, which report a positive relation between the severity of trauma exposure and level of reported post-traumatic growth [43, 44]. This effect is known to be strongest if people felt a substantial danger to their life and relates well to our findings that “Realizing the worth of life” and “Not taking health for granted” are two of the aspects of our questionnaire with the most positive answers [34]. This effect might be further emphasized by the perceived physical recovery over time, which could feel more substantial in people with more severe injuries.

## Strengths

Treatment of the patients’ injuries were performed exclusively at the same institution, facilitating a homogeneity in treatment strategies and quality of care. The number of included patients (*n* = 337) and the length of follow-up (20 years and longer) are substantial and post-traumatic growth after severe injury has not yet been investigated years after the injury. Additionally, as previous studies on this patient collective have already investigated medical, socio-economic and negative psychosocial sequelae, we are able to view our results in a more comprehensive context.

### Limitations

Data collection was performed through a self-administered questionnaire. As no additional psychological examinations have been performed, we cannot say with certainty whether perceived personal growth is associated with better psychological adjustment after injury. It also does not rule out the possibility of other psychological confounders. We would also like to point out, that a certain response bias cannot be ruled out. However, there are very similar patient characteristics in the group that could not be contacted. Also, there are previous publications on the same population, which focus on negative sequelae. Furthermore, we did neither investigate the effect of traumatic brain injury (TBI), nor correlation with post-traumatic stress disorder [35, 44]. PTDS has been shown to play a significant role in the report of post-traumatic growth [18]. Furthermore previous studies identified TBI as a predictor for inferior long-term psychological functioning and higher rates of chronic pain, which are likely to play a role in the development of PTG [7].

## Conclusion

Long-term post-traumatic growth in polytraumatized patients is a common development, which is encouraged by female gender, being married, older age, and higher injury severity. Emphasizing social support, personal relationships, appreciation of health and self-efficacy could positively influence this development. Further research on post-traumatic growth after severe injury and interventions to encourage its development are needed.
